# P095: The 2nd/3rd generation of cephalosprines use in antimicrobial stewardship program and contemporary carriage of E. coli and K. pneumoniae with esbls from identified or suspected infection hospitalized patients

**DOI:** 10.1186/2047-2994-2-S1-P95

**Published:** 2013-06-20

**Authors:** B Gao, L-H Zhao, H-Z Wu, G-D Wang, Z-H Guo, J-G Dai, W Lu

**Affiliations:** 1 Nosocomial Infection Control Unit, Tianjin Second People's Hospital, Tianjin, China; 2 Pharm Unit, Tianjin Second People's Hospital, Tianjin, China; 3 Clinical Microbiological Lab, Tianjin Second People's Hospital, Tianjin, China; 4 Graduate School, Tianjin Medical University, Tianjin, China; 5 Tianjin Second People's Hospital, Tianjin, China

## Objectives

To implement antimicrobial reasonable use and evaluate its impact on *E. coli* and *K. pneumoniae* with ESBLs isolated from hospitalized patients.

## Methods

The 2^nd^/3^rd^ generation cephalosprines (GCPs) use from 2006.1 to 2012.12 was converted to DDDs per 1,000 patient-days in a tertiary teaching infectious disease hospital in Tianjin, China. Multi-strategy, including contextually appropriate antibiotic use guidelines, active audit, as well as performance feedback/education in-field, were conducted by antimicrobial stewardship in the hospital since 2006. *E. coli* and *K. pneumoniae* isolated from all identified and suspected infections were selected at the same time. For ESBLs status, agar diffusion test with clavulanic acid plus or minus cefotaxime and ceftazidime was used. Linear correlation was carried out to analyze the relationship between 2^nd^/3^rd^ GCPs use and major Enterobacteriaceae producing ESBLs.

## Results

The 2^nd^ GCPs consumption was 12.488, 12.563, 12.637, 20.221, 48.598, 6.647, and 8.129 DDDs per 1,000 patient-days during 2006 to 2012 respectively*.* The 3^rd^ GCPs consumption was 35.248, 32.135, 31.720, 23.661, 31.696, 20.284 and 15.722 DDDs per 1,000 patient-days respectively at the same time*.* ESBL-producing strains rates of *E. coli* and *K. pneumoniae* were 27.42% (17/62), 26.79% (15/56), 29.09% (16/55), 23.53% (24/102), 39.29% (66/168), 25.58% (33/129) and 11.46% (18/157) from 2006 to 2012 respectively*.* Lower 2^nd^/3^rd^ GCPs annually use was associated with a lower rate of *E. coli* and *K. pneumoniae* with ESBLs isolated from contemporary hospitalized patients. (*r*^2^ = 0.843; *P* < 0.005).

## Conclusion

Multi-strategy intervention by antimicrobial stewardship was effective on commonly used 2^nd^/3^rd^ GCPs in the hospital of Tianjin, China. The decrease of 2^nd^/3^rd^ GCPs represented a significant driver in the reduction in rate of *E. coli* and *K. pneumoniae* with ESBLs from identified and suspected infections.

## Disclosure of interest

None declared

